# Enhancing the bioavailability of *Phyllanthus amarus*: implications for traditional and modern medicine in Africa

**DOI:** 10.1097/MS9.0000000000003514

**Published:** 2025-06-21

**Authors:** Arinze Favour Anyiam, Musa Abidemi Muhibi, Onyinye Cecilia Arinze-Anyiam, Emmanuel Ifeanyi Obeagu

**Affiliations:** a Department of Medical Laboratory Science, Faculty of Applied Health Sciences, Edo State University, Uzairue, Edo State, Nigeria; b Department of Medical Laboratory Science, School of Basic Medical and Health Sciences, Igbinedion University, Okada, Edo State, Nigeria; c Department of Biomedical and Laboratory Science, Africa University, Mutare, Zimbabwe

**Keywords:** Africa, bioavailability, modern medicine, Phyllanthus amarus, traditional medicine

## Abstract

*Phyllanthus amarus* has been a cornerstone of traditional medicine in Africa, renowned for its hepatoprotective, antiviral, and antidiabetic properties. However, its therapeutic applications in modern medicine are limited by poor bioavailability due to challenges such as low solubility, rapid metabolism, and instability of its bioactive compounds. Addressing these limitations is essential for optimizing its pharmacological potential and enabling its broader integration into evidence-based healthcare systems. This review highlights various strategies to enhance the bioavailability of *Phyllanthus amarus*, including nanotechnology, phytosome technology, biopiperine supplementation, fermentation, and sustained-release formulations. These approaches not only improve solubility and absorption but also stabilize the herb’s phytochemicals, ensuring better therapeutic outcomes. Moreover, the review explores how bioavailability enhancement can transform *Phyllanthus amarus* into a standardized therapeutic agent, bridging the gap between traditional and modern medicine. Enhancing the bioavailability of *Phyllanthus amarus* has profound implications for healthcare in Africa. Improved formulations can amplify its therapeutic efficacy in traditional practices, while enabling its adoption in modern pharmacology through clinical validation and pharmaceutical-grade products. By addressing cost, accessibility, and regulatory challenges, these advancements can ensure that *Phyllanthus amarus* continues to be a vital, sustainable resource in addressing pressing health issues across the continent.

## Introduction

*Phyllanthus amarus* is a medicinal plant renowned for its diverse therapeutic applications, particularly in traditional medicine. Research highlights its antioxidant properties, hepatoprotective effects, and the presence of various bioactive compounds, making it a subject of interest for both traditional and modern medicine. *P. amarus* exhibits significant antioxidant activity, with studies showing a DPPH inhibition of up to 74.4% at optimal concentrations^[1]^. The total phenolic content (TPC) and total flavonoid content (TFC) are notably high, indicating its potential as a natural antioxidant source^[1,2]^.The plant has demonstrated efficacy in protecting against liver damage, particularly in cases of gentamicin-induced hepatotoxicity in animal models^[3]^. Extracts have been shown to improve lipid profiles and reduce oxidative stress markers, suggesting a protective role against liver diseases^[3,4]^.*P. amarus* contains a variety of bioactive compounds, including flavonoids, lignans, and terpenoids, which contribute to its medicinal properties^[4,5]^.These compounds have been linked to both antioxidant and hepatoprotective activities, supporting its use in traditional remedies^[4]^.

**HIGHLIGHTS**

- Traditional significance: *Phyllanthus amarus* is widely used in African traditional medicine for its hepatoprotective, antiviral, and antidiabetic properties.
- Bioavailability challenges: Low solubility, rapid metabolism, and phytochemical instability limit its therapeutic potential in modern medicine.
- Enhancement strategies: Nanotechnology, phytosomes, piperine supplementation, and fermentation significantly improve absorption and stability.
- Modern medicine integration: Bioavailability advancements enable clinical validation and pharmaceutical standardization for broader adoption.
- Healthcare impact: Enhanced formulations can bridge traditional and modern medicine, promoting sustainable health solutions in Africa.

## Aim

The aim of this review is to critically explore and synthesize current knowledge on strategies for enhancing the bioavailability of *Phyllanthus amarus*, a plant with deep roots in African traditional medicine, and to evaluate how such advancements could bridge traditional practices with modern healthcare systems.

## Review methods

In developing this review on enhancing the bioavailability of *Phyllanthus amarus* and its implications for traditional and modern medicine in Africa, a systematic yet narrative-driven approach was adopted. The process began with a comprehensive literature search aimed at capturing the breadth of existing knowledge on *P. amarus*, its bioactive compounds, pharmacokinetics, and strategies for improving herbal bioavailability. Relevant scientific databases, including PubMed, Scopus, Google Scholar, and ScienceDirect, were searched using specific keywords such as “*Phyllanthus amarus*,” “bioavailability enhancement,” “nanotechnology and herbal medicine,” “traditional medicine in Africa,” and “phytochemical standardization.” Articles published between 2000 and 2025 were prioritized to ensure both historical context and contemporary insights were included. Studies focusing on the pharmacology, delivery systems, and clinical or preclinical evaluations of *P. amarus* were carefully reviewed. To maintain relevance and quality, preference was given to peer-reviewed journal articles, systematic reviews, meta-analyses, ethnobotanical reports, and clinical trial summaries. Grey literature, including theses and conference proceedings, was selectively used when they provided unique or region-specific insights, particularly about traditional African medicinal practices involving *P. amarus*.

Information was extracted and synthesized thematically. First, an overview of the pharmacologically active constituents of *P. amarus* was presented. This was followed by a detailed exploration of the challenges associated with its bioavailability. Subsequently, various strategies to overcome these barriers were organized into key categories such as nanotechnology applications, co-administration with bioenhancers, and novel delivery systems. Emphasis was placed on discussing not only the scientific underpinnings of these strategies but also their potential feasibility within the African context. Throughout the review process, an integrative lens was maintained, seeking to bridge traditional knowledge with modern biomedical advancements. Care was also taken to critically assess the robustness of the evidence, highlighting areas where data were strong as well as identifying gaps that require further research. While every effort was made to ensure a broad and balanced perspective, it is acknowledged that the dynamic and evolving nature of research in this field means that some emerging studies may not have been captured. Nevertheless, the methods employed aimed to produce a comprehensive and thoughtful narrative that informs both current understanding and future directions for the improved application of *Phyllanthus amarus* in healthcare systems across Africa.

## Review limitations

While this review provides an extensive overview of strategies for enhancing the bioavailability of *Phyllanthus amarus* and explores their implications for traditional and modern medicine in Africa, certain limitations must be acknowledged. First, although numerous preclinical studies suggest promising techniques for improving bioavailability, there is a notable scarcity of clinical trials specifically focused on enhanced formulations of *P. amarus*. As a result, much of the discussion remains theoretical or extrapolated from broader research on phytomedicines, rather than being grounded in direct clinical evidence. This gap limits the immediate applicability of some of the strategies discussed. Additionally, the review heavily relies on data generated from in vitro and animal models, which may not fully predict human responses. Factors such as metabolism, immune system variations, and gut microbiota differences between species could significantly influence the absorption and therapeutic outcomes of *P. amarus* in human populations. Thus, while novel delivery systems and bioenhancement strategies show potential, their translation to real-world clinical success remains uncertain without further validation. Another limitation lies in the geographic and cultural diversity of traditional *P. amarus* use across Africa. Practices, formulations, and methods of administration vary widely between regions and ethnic groups, making it difficult to propose a universally applicable enhancement strategy. The review attempts to provide a general perspective, but deeper, localized ethnomedical studies would be necessary to tailor bioavailability enhancement approaches to specific communities without undermining traditional knowledge systems.

Moreover, economic and technological feasibility is an important consideration that could not be fully explored in this review. While nanotechnology-based systems and advanced delivery methods offer exciting possibilities, their cost-effectiveness, scalability, and accessibility in resource-limited African settings remain challenging. Without parallel efforts in capacity building and infrastructure development, these innovations might struggle to achieve widespread adoption. Although the review highlights the potential socio-economic benefits of enhancing *P. amarus* bioavailability, it does not deeply delve into the policy, regulatory, and intellectual property issues that could impact the integration of improved traditional medicines into formal healthcare systems. Addressing these complex factors will be crucial for ensuring that the advancement of *P. amarus* formulations truly benefits local communities and supports sustainable healthcare development.

### Importance of bioavailability in determining pharmacological efficacy

The importance of bioavailability in determining pharmacological efficacy cannot be overstated, as it directly influences the therapeutic outcomes of drug therapies. Bioavailability refers to the proportion of a drug that enters systemic circulation and is available at the site of action, which is critical for achieving the desired therapeutic effects. Understanding and enhancing bioavailability is essential for optimizing drug efficacy and safety, particularly given the challenges posed by poor solubility and extensive first-pass metabolism in many pharmaceuticals. Bioavailability is defined as the rate and extent of drug absorption into systemic circulation^[2]^. Accurate measurement is vital for assessing a drug’s therapeutic potential. Various factors influence bioavailability, including solubility, stability, and the drug’s ability to penetrate biological membranes^[6]^. For instance, over 90% of drugs face limitations in oral bioavailability due to poor solubility^[7]^. Numerous strategies exist to improve bioavailability, such as size reduction, use of solubilizing excipients, and innovative drug delivery systems like nanotechnology^[7,8]^. These methods aim to increase the drug’s solubility and absorption efficiency. Ensuring adequate bioavailability is crucial for achieving therapeutic plasma concentrations, which directly impacts treatment efficacy and patient safety^[6]^.

### *Importance of* Phyllanthus amarus *in African traditional medicine*

*P. amarus* is widely recognized for its ability to protect the liver, making it a key remedy for jaundice and hepatitis^[9,10]^.The plant exhibits antidiabetic properties and is effective against various microbial infections, addressing conditions like diarrhea and dysentery^[10,11]^.It is employed in treating intermittent fevers, kidney disorders, and skin ailments, showcasing its versatility in traditional medicine^[9,10]^.In many African cultures, *P. amarus* is integral to herbal remedies, reflecting its deep-rooted significance in local health practices^[12]^.

### *Bioavailability and significance of* Phyllanthus amarus

*P. amarus* contains various bioactive compounds such as quercetin, geraniin, and rutin, which contribute to its medicinal properties^[9]^. The herb’s extracts have shown the ability to modulate oxidative stress and inflammatory pathways, particularly through the inhibition of TNF-α and interleukins^[13,14]^.

## Health benefits

Studies indicate that *P. amarus* can restore liver integrity and function, particularly in conditions of oxidative stress and ischemia^[14]^. The plant has demonstrated efficacy against diabetes and viral infections, notably hepatitis B^[9]^.

### Objective of the review

To evaluate the bioavailability of *P. amarus* in Africa. This involves examining the factors influencing its bioavailability, pharmacokinetics, and therapeutic applications, with a focus on how these aspects affect its efficacy and integration into both traditional and modern medicine in Africa.

### *Description of* Phyllanthus amarus *and its distribution in Africa*

The bioavailability of *Phyllanthus amarus* in Africa is supported by its diverse pharmacological properties and traditional uses, particularly in treating various ailments.

*Phyllanthus amarus* contains various bioactive compounds, including alkaloids, phenols, saponins, and tannins, which contribute to its medicinal properties^[15]^. These compounds are responsible for its antioxidant, anti-inflammatory, and antimicrobial activities, making it a valuable resource in traditional medicine. Studies demonstrate that the ethanol extract of *Phyllanthus amarus* effectively reduces parasitaemia in trypanosomiasis, improving clinical parameters in infected rats^[15]^. Additionally, its aqueous extract has shown antihypertensive effects and improved cardiac function in hypertensive models^[16]^.

### *Summary of reported pharmacological activities of* Phyllanthus amarus

*Phyllanthus amarus* exhibits significant antiviral properties, particularly against HIV. Extracts from the plant have been shown to inhibit HIV-1 attachment and the activity of key enzymes such as integrase and reverse transcriptase, demonstrating effectiveness both *in vitro* and *in vivo*^[17]^.The plant has broad-spectrum antibacterial effects against both gram-positive and gram-negative bacteria. It has been found to inhibit drug-resistant strains, making it a potential candidate for treating bacterial infections^[18,19]^.*P. amarus* is noted for its hepatoprotective properties, helping to restore liver function and protect against liver damage from toxins such as alcohol and certain medications^[20]^.Research indicates that extracts of *P. amarus* can lower blood glucose levels and improve insulin sensitivity, making it beneficial for managing diabetes^[21]^.The herb has shown promise in inhibiting cancer cell proliferation and inducing apoptosis in various cancer models. Its extracts have been linked to the prevention of chemically induced tumors and may possess anti-mutagenic properties^[19,20]^.*Phyllanthus amarus* has been reported to reduce inflammation and pain, contributing to its use in traditional medicine for treating various inflammatory conditions^[20]^. Studies have demonstrated that *P. amarus* can protect against nephrotoxicity induced by drugs like gentamicin, helping to maintain kidney function^[18-22]^. The plant’s extracts are rich in antioxidants, which help combat oxidative stress and may contribute to its overall health benefits^[20]^.Table 1 shows summary of key bioactive compounds in *Phyllanthus amarus*^[23-27]^.

**Table 1:** Summary of key bioactive compounds in *Phyllanthus amarus*

Component class	Key compounds	Pharmacological activities
Lignans^[19,20]^	Phyllanthin, hypophyllanthin, niranthin, nirtetralin, phyltetralin	Hepatoprotective, antiviral, anticancer, antioxidant
Flavonoids^[20,22]^	Various subclasses like flavanones, quercetin, rutin	Antioxidant, anti-inflammatory
Alkaloids^[23]^	Various	Antimicrobial, antidiabetic
Terpenes^[19]^	Lupeol, oleanolic acid	Anti-inflammatory, antimicrobial
Tannins^[19]^	Amariin, ellagitannin, geraniin, corilagin, 1,6-digalloylglucopyranoside, rutin, quercetin-3-0-glucopyranoside	Antibacterial, antioxidant
Phenolic compounds^[24]^	Phyllanthusiin D, amariin, repansunic acid	Antioxidant
Other compounds^[24]^	Saponins, cardiac glycosides, phytol, hexadecanoic acid, ethyl ester, β-tocopherol, benzenamine,N-[2-(3,4-dimethoxyphenyl) thyl]-2-nitro-, 9,12,15-octadecatrienoic acid, 1-methyl ester, (Z,Z,Z)-	Immune modulation, cardiovascular effects, anti-inflammatory, hypo-cholesterolemic, cancer preventive, hepato-protective, bactericidal, antioxidant, diuretic, antitumor, antimicrobial

### *Pharmacokinetic profiles of* P. amarus *reported in global studies*

*Phyllanthus amarus* has garnered attention in pharmacological research for its potential herb-drug interactions and its impact on drug metabolism. Various studies have explored the pharmacokinetic profiles of this plant, particularly focusing on its effects on specific drugs and metabolic pathways. A study investigated the effect of *Phyllanthus amarus* extract on the pharmacokinetics of midazolam (MDZ), a substrate for cytochrome P450 3A (CYP3A) enzymes. Oral administration of *P. amarus* extract significantly increased the maximum concentration (Cmax) and area under the curve (AUC) of MDZ, with increases of 3.9-fold and 9.6-fold, respectively, while decreasing the clearance by 12% after a single dose. This suggests that *P. amarus* can inhibit intestinal CYP3A, enhancing oral bioavailability of MDZ^[27-29]^. In a study involving daily administration of *P. amarus* extract over 15 days, it was found to induce the activity and expression of CYP3A and CYP2B1/2 enzymes in rats. This dual action indicates that while acute exposure may inhibit certain metabolic pathways, chronic exposure could lead to an induction effect, potentially altering the metabolism of co-administered drugs^[30-32]^.

A pharmacokinetic on *Phyllanthus amarus* revealed that its 80% ethanolic extract significantly inhibited the production of pro-inflammatory mediators such as TNF-α, IL-1β, and PGE2 in LPS-induced U937 human macrophages. The extract also downregulated COX-2 protein expression and the mRNA transcription of pro-inflammatory markers in a dose-dependent manner. Furthermore, it was found to suppress the phosphorylation of key signaling molecules involved in inflammation, including NF-κB, Akt, and various MAPKs (JNK, ERK, and p38). Additionally, *P. amarus* extract reduced the expression of upstream signaling molecules TLR4 and MyD88, which are crucial for activating NF-κB, MAPK, and PI3K-Akt pathways. The quantitative analysis identified significant levels of lignans and polyphenols in the extract, suggesting that these compounds may contribute to its anti-inflammatory effects^[33-35]^.Additional research has indicated that *P. amarus* may also inhibit other cytochrome P450 enzymes such as CYP1A2, CYP2C9, and CYP2D6, which are crucial for phase I drug metabolism. This inhibition could lead to significant herb-drug interactions when *P. amarus* is co-administered with medications metabolized by these enzymes^[36,37]^. The findings suggest that co-administration of *P. amarus* with drugs metabolized by CYP3A and other affected enzymes could result in therapeutic failure or adverse effects due to altered drug metabolism.

### Therapeutic applications, with emphasis on conditions prevalent in Africa

*Phyllanthus amarus* has shown promising antimalarial properties, particularly against *Plasmodium falciparum*, the causative agent of malaria. Studies indicate that both ethanolic and aqueous extracts exhibit significant reductions in parasite growth, with the ethanolic extract demonstrating an inhibition rate of 76.8% and a half-maximal inhibitory concentration (IC50) of 5.80 μg/ml^[38]^. The plant is often prepared as a decoction, with traditional healers recommending its use for malaria treatment alongside other herbal remedies to enhance efficacy^[39]^.*P. amarus* is traditionally used to treat liver-related ailments such as jaundice and hepatitis. Its bioactive compounds have been linked to hepatoprotective effects, helping to safeguard liver cells from damage caused by toxins and infections^[40]^. The presence of lignans like phyllanthin contributes to these protective effects, making it valuable in managing liver diseases prevalent in many African communities. The plant is also recognized for its antidiabetic effects, with studies suggesting that it can lower blood glucose levels and enhance insulin sensitivity^[41]^. Given the high prevalence of diabetes in Africa, *P. amarus* offers a potential natural remedy for managing this chronic condition and other ailments like gonorrhea, diarrhea and dysentery, respiratory issues like asthma, and skin ulcers^[42]^.

## Factors affecting bioavailability of *P. amarus* in Africa

The mineral content and overall quality of the soil where *P. amarus* is grown can impact the nutrient composition of the plant. Poor soil conditions may lead to lower concentrations of bioactive compounds such as lignans (phyllanthin and hypophyllanthin), which are critical for its pharmacological effects^[43]^. Temperature, rainfall, and humidity levels significantly influence the growth and biosynthesis of active compounds in *P. amarus*. Optimal conditions (20-35 °C) are necessary for effective germination and growth, which directly affects the plant’s medicinal properties^[30]^.The method used to prepare extracts from *P. amarus* (e.g., aqueous, ethanol) can significantly influence the concentration and bioavailability of its active compounds. For instance, 50% ethanol extracts have shown effective antibacterial properties, indicating that solvent choice impacts the extraction efficiency of bioactive components^[28]^. Traditional methods of preparing herbal remedies may vary widely across regions and can affect the stability and bioavailability of active ingredients. Cooking or decocting the plant material may enhance or degrade certain compounds. The existing nutritional status of individuals consuming *P. amarus* can influence how well they absorb its active compounds. For example, individuals with deficiencies in specific nutrients may have altered absorption rates for certain phytochemicals present in the herb.

The metabolism of bioactive compounds from *P. amarus* can vary based on individual differences in liver enzyme activity (e.g., cytochrome P450 enzymes). This variability can lead to differences in how effectively these compounds exert their therapeutic effects^[28]^. Co-administration with other medications may alter the pharmacokinetics of *P. amarus* constituents, either enhancing or inhibiting their absorption and efficacy. The availability and quality of *P. amarus* may be affected by socioeconomic factors such as agricultural practices, market access, and conservation efforts. Poor harvesting practices can lead to degradation or contamination of plant materials. Knowledge about proper preparation methods and dosages among users can influence the effective use of *P. amarus* in traditional medicine. Table 2 shows challenges in bioavailability studies of *Phyllanthus amarus*^[27,31,32]^. Table 3 shows current bioavailability enhancement strategies^[33-36]^.

**Table 2:** Challenges in bioavailability studies of *Phyllanthus amarus*

Challenge	Description
Limited water solubility^[31]^	Key bioactive components like lignans and flavonoids have poor water solubility, restricting their absorption and leading to reduced bioavailability, making it hard to achieve therapeutic concentrations in the bloodstream.
Extraction efficiency^[27]^	The choice of extraction solvent affects the yield and potency of active compounds. Polar solvents may not extract sufficient lipophilic compounds, potentially resulting in suboptimal therapeutic effects.
Variability in traditional practices^[32]^	Preparation methods for *P. amarus* vary widely across cultures, leading to inconsistencies in active ingredient concentrations, complicating comparisons across studies and limiting standardized dosages.
Crude extracts vs. isolated compounds^[32]^	Many studies focus on crude extracts without detailed chemical characterization, making it difficult to correlate bioactivity with specific compounds and hindering understanding of pharmacokinetics.
Limited animal and human trials^[27]^	Most research involves in vitro studies, which do not replicate complex interactions in living organisms, restricting insights into absorption, distribution, metabolism, and excretion (ADME) processes crucial for assessing bioavailability.
Individual differences^[27]^	Variability among individuals due to genetic factors, health status, and concurrent medications can affect metabolism and utilization of *P. amarus*, complicating predictions of therapeutic outcomes based on standard dosages.

**Table 3:** Current bioavailability enhancement strategies

Bioavailability strategy	Description
Nanotechnology approaches^[33,34]^	Nanoparticles and nanocarriers: utilize nanoparticles like liposomes and solid lipid nanoparticles (SLNs) to enhance solubility and stability. Nanoemulsions and nanosuspensions: increase surface area for improved dissolution and bioavailability, especially for hydrophobic drugs.
Drug delivery systems^[35]^	Self-emulsifying drug delivery systems (SEDDS): promote emulsions upon contact with gastrointestinal fluids, enhancing solubility and absorption of lipophilic drugs.
	Microspheres and liposomes: encapsulate drugs to protect from degradation and improve solubility through controlled release.
Chemical modifications^[34]^	Prodrugs: modify drug structure to enhance solubility and permeability; prodrugs are metabolized into active forms in the body. Cyclodextrin complexation: form inclusion complexes with poorly soluble drugs to improve solubility and stability, particularly for oral bioavailability.
Solid dispersion techniques^[34]^	Disperse drugs within a polymer matrix to enhance dissolution rate, improving bioavailability of poorly soluble compounds.
Particle size reduction^[36]^	Micronization: reducing particle size increases surface area for dissolution, enhancing bioavailability of solid dosage forms.
	Nanosizing: further reduction to nanoscale for greater improvements in solubility and absorption.
Use of surfactants^[36]^	Incorporate surfactants to reduce surface tension and improve drug solubility; penetration enhancers facilitate transport across biological membranes.
Biochemical strategies^[35]^.	Targeted delivery systems: develop systems targeting specific tissues or cells to enhance efficacy and minimize side effects.
	Environment-sensitive particles: respond to pH or temperature changes, releasing payloads in specific environments (e.g., tumor sites) for improved therapeutic outcomes.

## Conclusion

Enhancing the bioavailability of *Phyllanthus amarus* is essential for maximizing its therapeutic potential and integrating its traditional use into modern medicine in Africa. Despite its rich pharmacological profile, factors such as poor solubility, variable preparation methods, and metabolic differences pose significant challenges to its efficacy. Advanced strategies like nanotechnology, self-emulsifying drug delivery systems, and chemical modifications offer promising solutions to overcome these barriers. By addressing these bioavailability challenges, *P. amarus* can become a more effective and reliable treatment option, bridging the gap between its traditional applications and contemporary medical practices in Africa.

## Data Availability

Not applicable as this a narrative review.

## Research registration unique identifying number (UIN)

Not applicable as this a narrative review.
